# Phylogenetic reconstruction using secondary structures and sequence motifs of ITS2 rDNA of *Paragonimus westermani *(Kerbert, 1878) Braun, 1899 (Digenea: Paragonimidae) and related species

**DOI:** 10.1186/1471-2164-10-S3-S25

**Published:** 2009-12-03

**Authors:** Pramod Kumar Prasad, Veena Tandon, Devendra Kumar Biswal, Lalit Mohan Goswami, Anupam Chatterjee

**Affiliations:** 1 Department of Zoology, North-Eastern Hill University, Shillong-793022, Meghalaya, India; 2 Bioinformatics Centre, North-Eastern Hill University, Shillong-793022, Meghalaya, India; 3 Department of Biotechnology & Bioinformatics, North-Eastern Hill University, Shillong-793022, Meghalaya, India

## Abstract

**Background:**

Most phylogenetic studies using current methods have focused on primary DNA sequence information. However, RNA secondary structures are particularly useful in systematics because they include characteristics that give "morphological" information, not found in the primary sequence. In several mountainous regions of Northeastern India, foci of *Paragonimus *(lung fluke) infection reportedly involve species that are known to prevail in neighbouring countries. The present study was undertaken to demonstrate the sequence analysis of the ribosomal DNA (ITS2) of the infective (metacercarial) stage of the lung fluke collected from the edible crab hosts that are abundant in a mountain stream of the area (Miao, Changlang District in Arunachal Pradesh) and to construct its phylogeny. Using the approach of molecular morphometrics that is based on ITS2 secondary structure homologies, phylogenetic relationships of the various isolates of *Paragonimus *species that are prevalent in the neighbouring Near-eastern countries have been discussed.

**Results:**

Initially, ten predicted RNA secondary structures were reconstructed and the topology based only on the predicted RNA secondary structure of the ITS2 region resolved most relationships among the species studied. We obtained three similar topologies for seven species of the genus *Paragonimus *on the basis of traditional primary sequence analysis using MEGA and a Bayesian analysis of the combined data. The latter approach allowed us to include both primary sequence and RNA molecular morphometrics; each data partition was allowed to have a different evolution rate. *Paragonimus westermani *was found to group with *P. siamensis *of Thailand; this was best supported by both the molecular morphometrics and combined analyses. *P. heterotremus*, *P. proliferus*, *P. skrjabini*, *P. bangkokensis *and *P. harinasutai *formed a separate clade in the molecular phylogenies, and were reciprocally monophyletic with respect to other species. ITS2 sequence motifs allowed an accurate in-silico distinction of lung flukes.

**Conclusion:**

Data indicate that ITS2 motifs (≤ 50 bp in size) can be considered a promising tool for trematode species identification. RNA secondary structure analysis could be a valuable tool for distinguishing new species and completing *Paragonimus *systematics, more so because ITS2 secondary structure contains more information than the usual primary sequence alignment.

## Introduction

The lung flukes of the genus *Paragonimus *have been known as one of the most important zoonotic parasites causing paragonimiasis, also known as endemic haemoptysis, in man. It is estimated that over 20 million people are infected worldwide due to several species of *Paragonimus *[1]. Over 40 species are known to infect the lung of different mammalian hosts (representing as many as eleven families) throughout the world [2] and approximately 15 species are known to infect humans. The parasite can migrate to several other vital tissues including brain [3]. The best known species is *P. westermani *[4] Braun, 1899 - a human parasite that can undergo development in as many as 16 different snail species and 50 crustacean species. Beside *P. westermani*, several other species namely, *P. pulmonalis *(Baelz, 1880) Miyazaki, 1978; *P. ohirai *Miyazaki, 1939; *P. iloktsunensis *Chen, 1940; *P. skrjabini *Chen, 1959; *P. miyazaki *Kamo et al., 1961 and *P. heterotremus *Chen and Hsia, 1964 - all reported to be occurring in Asia; *P. africanus *and *P. uterobilateralis *Voelker and Vogel, 1965 in Africa; and *P. mexicanus *Miyazaki and Ishii, 1968 in America are considered pathogenic to man. While *P. westermani *is distributed mostly in Asia, *P. heterotremus *is the predominant causative agent of paragonimiasis in Thailand [5].

In the context of India, the states of West Bengal, Assam and some other parts of the country are endemic foci of human paragonimiasis. This infection has been reported in a sizeable human population of Manipur, a north-eastern state of India [6,7]. Very recently in Manipur and Arunachal Pradesh (Northeast India), the suspected foci of human infection where consumption of crustacean intermediate hosts is of regular practice, the Chinese species, *P. hueitungensis *and *P. heterotremus*, respectively were identified as etiological agents of paragonimiasis [8,9]. However, no or scanty information is available about the prevalence of the parasite among its molluscan and crustacean intermediate hosts as even in the suspected foci of human infection.

Morphology of the encysted and excysted metacercariae, which occur as the infective stage in the muscle tissue of the crustacean second intermediate host, has been conventionally used in identification of species of *Paragonimus*. The taxonomy of *Paragonimus *spp has been based mainly on morphological data complemented with ecological, cytological and pathological results as well as clinical manifestations. Morphological differences found on stained and mounted adult specimens have been widely used to discriminate between platyhelminth species. Thus, identification of closely related metazoan parasite species based on morphological characters alone can be difficult. In recent times, the amplification of specific DNA regions via the polymerase chain reaction (PCR) and improved sequencing techniques have been employed to resolve taxonomic issues related to various helminth parasites by comparing their DNA. PCR-based techniques utilizing the rDNA ITS2 sequences, which occur between the 5.8S and 28S coding regions, have proven to be a reliable tool to identify the helminth species and their phylogenetic relationships [10-12]. Studies on phylogeny and/or intraspecific variation in *Paragonimus *species have also been done using ITS2 region [13,14].

Most phylogenetic studies using current methods have focused on primary DNA sequence information. However, RNA secondary structures are particularly useful in systematics because they include characteristics, not found in the primary sequence, that give 'morphological' information. The molecular morphometrics approach has been employed in the present study for comparisons between primary and secondary structure information. It is an established fact that rRNA structure is highly conserved throughout evolution as most of the folding is functionally important despite primary sequence divergence [15]. The novel approach of molecular morphometrics that relies both on traditional morphological comparison and on molecular sequence comparison by measuring the structural parameters of the ITS2 secondary structure homologies (geometrical features, bond energies, base composition etc.) is recently being used to study the phylogenetic relationships of various species [16]. It is well known that rRNA is highly conserved throughout evolution. Thus, the secondary-structure elements of the RNA molecule, i.e., the helices, loops, bulges, and separating single-stranded portions, can be considered phylogenetic characters [17-19].

The present study was undertaken to demonstrate the sequence analysis of the ribosomal DNA (ITS2) of the infective metacercarial stages of the lung fluke, which abound in the muscle tissue of the edible crab hosts abundant in mountain streams of a suspected focus of infection and to construct its phylogeny using rRNA secondary structures supplementing the primary sequence analysis.

## Methods

### Parasite material and DNA isolation, amplification and sequencing

Naturally infected freshwater edible crabs (*Barytelphusa lugubris*) were collected from a mountain stream of Miao, Changlang District in Arunachal Pradesh (Altitude - 213 mASL, Longitude - 96°-15'N and Latitude - 27°-30'E). Metacercariae were isolated from the muscles of the crustacean host by digestion technique in artificial gastric juice. The sediments were examined for *Paragonimus *metacercariae under a dissecting stereoscopic microscope.

For the purpose of DNA extraction, metacercariae collected from the same part of stream were pooled. The 70% alcohol-fixed metacercariae were further processed for DNA extraction and PCR amplification following the earlier standardised procedure and protocol. The rDNA region spanning the ITS2 was amplified from metacercarial DNA by PCR; using trematode universal primers designed based on *Schistosoma *species [20].

**3S **(forward): 5'GGTACCGGTGGATCACTCGGCTCGTG-3'

**A28 **(reverse): 5'-GGGATCCTGGTTAGTTTCTTTTCCTCCGC-3'

The PCR amplification was performed following the standard protocol with minor modifications as described earlier [21].

### Molecular phylogenetic analysis using bioinfomatic tools

The DNA sequences were put to further analysis with the usage of bioinfomatics tools including similarity search using BLAST (Basic Local Alignment Search Tool); http://www.ncbi.nlm.nih.gov/blast, and phylogenetic prediction using ClustalW provided at the http://www.ebi.ac.uk/clustalw for query DNA sequence. A total of 10 ITS2 sequences were selected (4 Indian isolates and 6 Isolates of Neighbouring countries) depending upon their BLAST hits and E-value. Only unique sequences were used in tree construction; ITS sequences arranged with MEGA format were entered in the MEGA [22] for construction of phylogenetic trees that were inferred using distance method like Neighbor Joining and character state method Maximum Parsimony. Test of phylogenetic accuracy was done by bootstrapping.

### Predicted ITS2 RNA secondary structures and analyses

Secondary structures of ITS2 sequences of various paragonimid species were reconstructed by aligning their sequences using Bioedit [23]. The acquired structures with restrictions and constrains were submitted in MFOLD [24]. RNA was folded at a fixed temperature of 37°C and the structure chosen from different output files was the desired 6-helicoidal ring or the one with the highest negative free energy if various similar structures were obtained.

### Motif identification, testing and validation

The ITS sequence motifs were identified from aligned sequences of the data set for the species using PRATT software http://www.ebi.ac.uk/Tools/pratt/index.html. The minimum percentage of sequences to match (C%) parameter was adjusted to report pattern matching at 100% of the sequence input. The motifs were expressed using the DNA alphabet (A, T, C, G) in PROSITE language [25]. The validation of the motifs was performed for each species using a "PATTERN MATCHING" Web application http://genoweb.univ-rennes1.fr/Serveur-GPO/. A motif was considered highly specific to a *Paragonimus *species if it matched most or all the ITS sequences of this species but no other ITS of any other trematode species.

### Bayesian phylogenetic analysis

DNA sequences were aligned using ClustalX 2.0.7. The interleaved NEXUS file was edited manually in order for it to be recognized by Mr. Bayes V3.1.2 programme. Phylogenetic analysis was carried out using the Bayesian approach with combined datasets (wherein each data partition is allowed to have a different evolution rate) using default parameters. The cladogram with the posterior probabilities for each split and a phylogram with mean branch lengths were generated and subsequently read by the tree drawing program Tree view V1.6.6.

## Results

### Construction of phylogenetic trees

**Figure 1:**
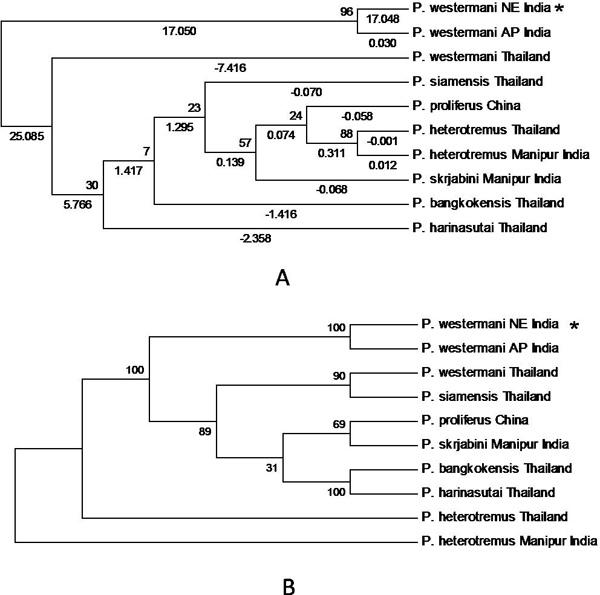
**Phylogenetic trees of ITS2 sequences of Paragonimid species showing bootstrap values**. (*) = Query sequence. **(A) **Neighbor Joining showing bootstrap values and distance. **(B) **Maximum Parsimony showing bootstrap values.

Phylogenetic trees were obtained by comparing the ITS2 sequences of *Paragonimus *species from different geographical isolates. Phylogenetic analyses using the NJ and MP methods showed that the topology is similar among the trees obtained (Fig. 1A &1B). The Evolutionary Divergence between sequences was also estimated and the number of base substitutions per site from analysis between sequences is shown. All results are based on the pairwise analysis of 10 sequences. All analyses were conducted in MEGA4.

### In-silico identification of *Paragonimus *species based on pattern matching ITS motifs

**Figure 2:**
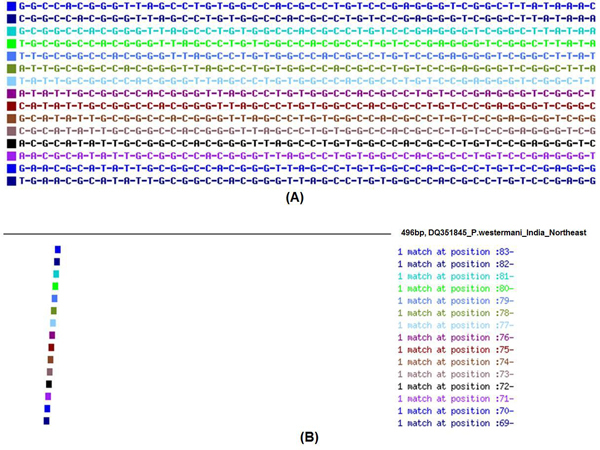
**(A) Sequence motif in PROSITE format (from 5' to 3' ends)**. **(B) **Pattern matching of ITS motifs of *P. westermani *India (496 bp).

A total of 10 ITS2 motifs were tested by BLAST analysis against the generalized GenBank database at NCBI (Table 1). All the motifs exhibited exact or perfect matches with that of the genus *Paragonimus *from different geographical isolates with 100% identity and significant E-value scores of 8e^-19 ^(Figs. 2A &2B).

**Table 1:** BLAST outputs of *Paragonimus *ITS sequence motifs against NCBI GenBank database (nr db)

Species motif patterns (5'-3' ends)	Length (bp)	No. of best hits	Identity (%)	E-value
>Pattern1 G-G-C-C-A-C-G-G-G-T-T-A-G-C-C-T-G-T-G-G-C-C-A-C-G-C-C-T-G-T-C-C-G-A-G-G-G-T-C-G-G-C-T-T-A-T-A-A-A-C	50	100	100	8e^-19^
>Pattern2 C-G-G-C-C-A-C-G-G-G-T-T-A-G-C-C-T-G-T-G-G-C-C-A-C-G-C-C-T-G-T-C-C-G-A-G-G-G-T-C-G-G-C-T-T-A-T-A-A-A	50	100	100	8e^-19^
>Pattern3 G-C-G-G-C-C-A-C-G-G-G-T-T-A-G-C-C-T-G-T-G-G-C-C-A-C-G-C-C-T-G-T-C-C-G-A-G-G-G-T-C-G-G-C-T-T-A-T-A-A	50	100	100	8e^-19^
>Pattern4 T-G-C-G-G-C-C-A-C-G-G-G-T-T-A-G-C-C-T-G-T-G-G-C-C-A-C-G-C-C-T-G-T-C-C-G-A-G-G-G-T-C-G-G-C-T-T-A-T-A	50	100	100	8e^-19^
>Pattern5 T-T-G-C-G-G-C-C-A-C-G-G-G-T-T-A-G-C-C-T-G-T-G-G-C-C-A-C-G-C-C-T-G-T-C-C-G-A-G-G-G-T-C-G-G-C-T-T-A-T	50	100	100	8e^-19^
>Pattern6 A-T-T-G-C-G-G-C-C-A-C-G-G-G-T-T-A-G-C-C-T-G-T-G-G-C-C-A-C-G-C-C-T-G-T-C-C-G-A-G-G-G-T-C-G-G-C-T-T-A	50	100	100	8e^-19^
>Pattern7 T-A-T-T-G-C-G-G-C-C-A-C-G-G-G-T-T-A-G-C-C-T-G-T-G-G-C-C-A-C-G-C-C-T-G-T-C-C-G-A-G-G-G-T-C-G-G-C-T-T	50	100	100	8e^-19^
>Pattern8 A-T-A-T-T-G-C-G-G-C-C-A-C-G-G-G-T-T-A-G-C-C-T-G-T-G-G-C-C-A-C-G-C-C-T-G-T-C-C-G-A-G-G-G-T-C-G-G-C-T	50	100	100	8e^-19^
>Pattern9 C-A-T-A-T-T-G-C-G-G-C-C-A-C-G-G-G-T-T-A-G-C-C-T-G-T-G-G-C-C-A-C-G-C-C-T-G-T-C-C-G-A-G-G-G-T-C-G-G-C	50	100	100	8e^-19^
>Pattern10 G-C-A-T-A-T-T-G-C-G-G-C-C-A-C-G-G-G-T-T-A-G-C-C-T-G-T-G-G-C-C-A-C-G-C-C-T-G-T-C-C-G-A-G-G-G-T-C-G-G	50	100	100	8e^-19^

### Secondary structure analysis

**Figure 3:**
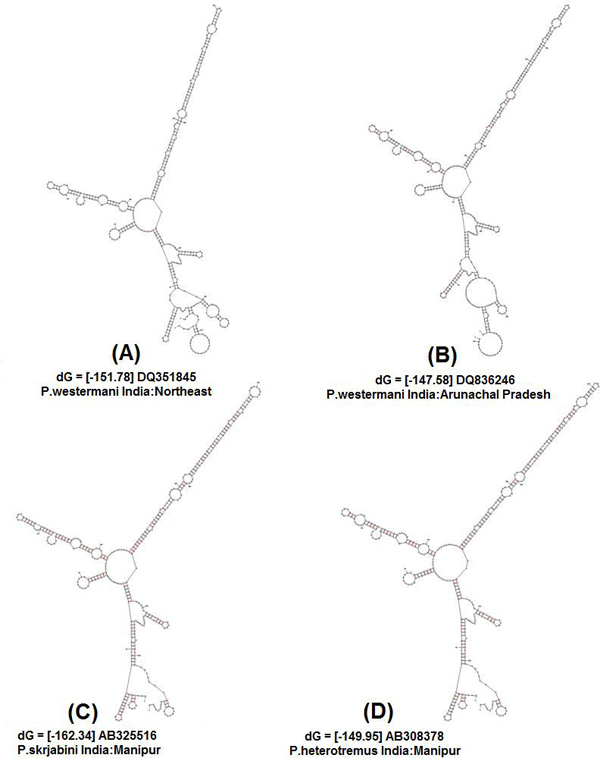
**Predicted ITS2 RNA secondary structures and their structure formation enthalpies according to MFOLD: Indian isolates**. **(A) ***P. westermani *India:Northeast, ΔG = -151.78 kcal/mole. **(B) ***P. westermani *India:Arunachal Pradesh, ΔG = -147.58 kcal/mole. **(C) ***P. skrjabini *India:Manipur, ΔG = -162.34. kcal/mole. **(D) ***P. heterotremus *India:Manipur, ΔG = -149.95 kcal/mole.

**Figure 4:**
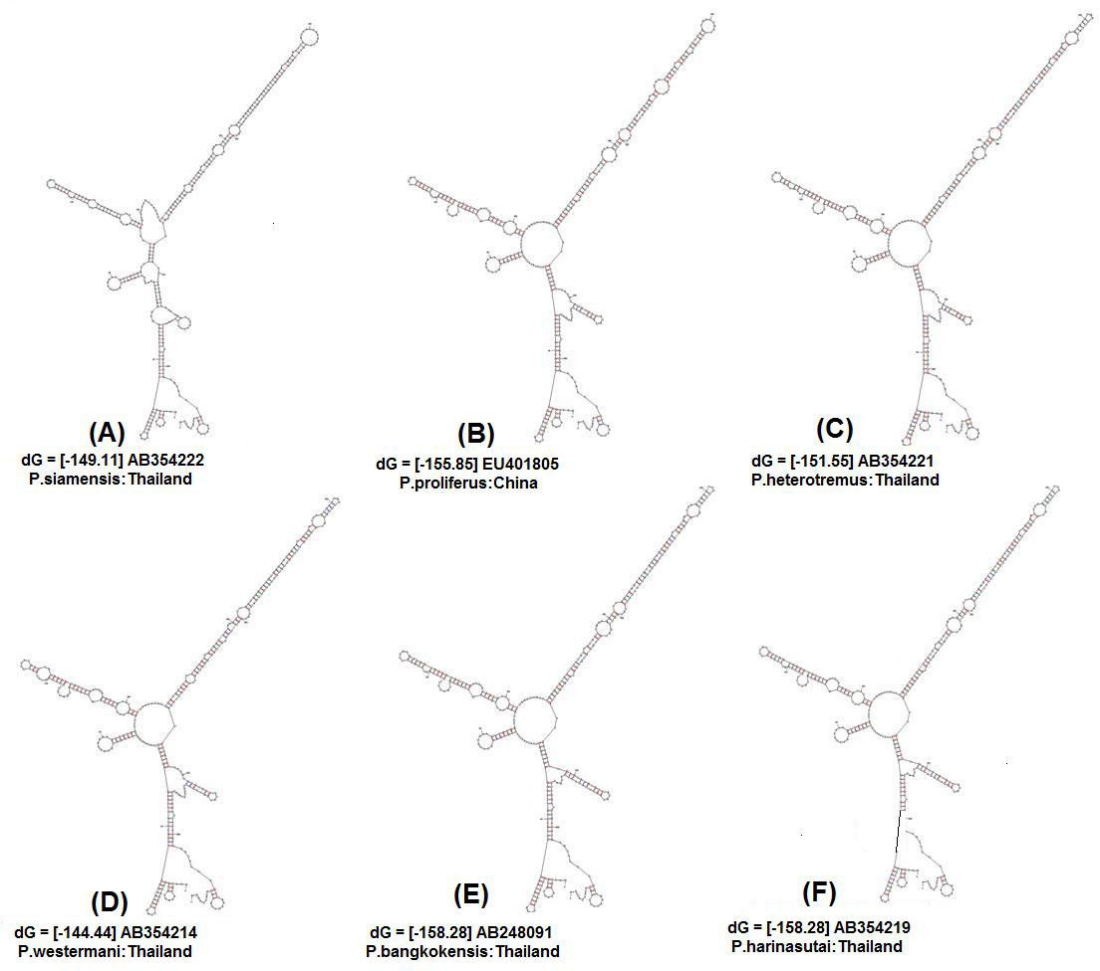
**Predicted ITS2 RNA secondary structures and their structure formation enthalpies according to MFOLD: Isolates from neighbouring countries**. **(A) ***P. siamensis *Thailand, ΔG = -149.11 kcal/mole. **(B) ***P. proliferus *China, ΔG = -155.85 kcal/mole. **(C) ***P. heterotremus *Thailand, ΔG = -151.55 kcal/mole. **(D) ***P. westermani *Thailand, ΔG = -144.44 kcal/mole. **(E) ***P. bangkokensis *Thailand, ΔG = -158.28 kcal/mole. **(F) ***P. harinasuti *Thailand, ΔG = -158.28 kcal/mole.

**Figure 5:**
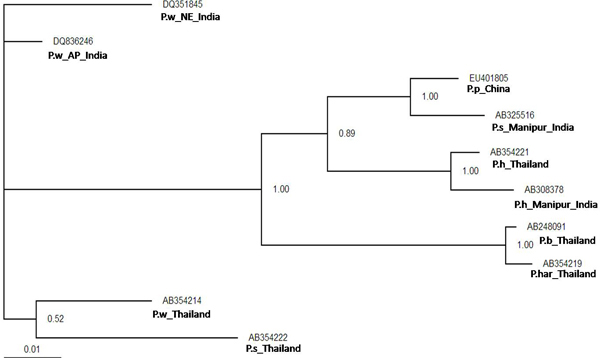
**Phylogenetic relationships between members of family Paragonimidae**. This tree shows hypothetical Bayesian analysis phylogeny based on the secondary structure alignment data of the ITS2 region. The numbers are equivalent to bootstrap percentages

Initially, ten predicted RNA secondary structures were reconstructed to provide the basic information for phylogenetic analyses; they accorded with the 6 helicoidal ring model (Figs. 3A-D &4A-F). The secondary structural features of ITS2 regions as shown in the figures were analysed based on conserved stems and loops, which in order of preference were interior loop, bulge loop, multiple branch loop, hairpin loop and exterior loop in all the isolates. We obtained three similar topologies for seven species of the genus *Paragonimus *on the basis of traditional primary sequence analyses using MEGA and a Bayesian analysis of the combined data (Fig. 5). The latter approach allowed us to include both primary sequence and RNA molecular morphometrics; each data partition was allowed to have a different evolution rate. *Paragonimus westermani *was found to group with *P. siamensis *of Thailand; this was best supported by both the molecular morphometrics and combined analysis. *P. heterotremus*, *P. proliferus*, *P. skrjabini*, *P. bangkokensis *and *P. harinasutai *formed a separate clade in the molecular phylogeny and were reciprocally monophyletic with respect to other species.

The ITS2 RNA secondary structures yielded homologous models that grouped conserved species features in the *Paragonimus *genus (e.g. long helix IV), though it is important to note that these molecular structures can also produce homoplasies because of selective pressures acting on particular helix features such as the 6-helicoidal ring core, conserved among most eukaryotes. This approach resulted in a supported Bayesian inference phylogram very similar to the molecular morphometrics approach. The major difference between the topologies obtained by the Bayesian (e.g. molecular morphometrics using partitions) and primary sequence methodologies was the placement of *P. siamensis *of Thailand, which could be due to the lack of phylogenetic signal and the likely saturation of ITS2 sequences after multiple alignments. However, *P. westermani*, grouping with *P. siamensis *of Thailand, showed an overall similarity in the ITS2 rRNA folding and identical secondary structures, which in the remaining five isolates showed some variation. Generally RNA secondary structure prediction programs rely on free energy minimization using nearest-neighbor parameters for predicting the overall structural stability in terms of Gibbs free energy at 37°C. The observed similarities at the secondary structural level are further reflected at the energy level (-ΔG). The difference in their topology, however, is due to differences in nucleotide sequence lengths. Moreover, the observed phylogenetic trend was identified with respect to the target accessibility sites for the seven different isolates. Bayesian analysis of the alignment retained the same topology and supported the same branches of the trees derived from the primary sequence data.

## Discussion

In sequence analysis of the rDNA ITS2, comparing with the known sequences of the other lung fluke species, the present study revealed that the sequence of ITS2 (plus flanking regions) show close resemblance with that of *Paragonimus westermani*. In phylogenetic analysis, as a general rule, if the bootstrap value for a given interior branch of a phylogenetic tree is 70% or higher, then the topology at that branch is considered reliable. Our results showed a bootstrap value to be >70% among the trees obtained and the ITS2 sequence resembled *Paragonimus westermani*, another Indian isolate [GenBank: DQ336246]. The comparison of ITS sequences from the parasites of different hosts and of different countries indicates that there exists a high species-specific homogeneity. In the present study, primary sequence analysis revealed a close relationship between the query sequence and other isolates from NER of India and those from neighbouring countries. Thus, on the basis of absolute matching of ITS2 sequence that could be used as one of the species markers, it can be concluded that *Paragonimus *species prevailing in Miao region of Arunachal Pradesh is indeed *P. westermani *and not *P. heterotremus *that was earlier reported from the same host and locality [9].

In phylogenetic studies involving secondary structure analysis as a tool, RNA folding is used for refining the alignment. Although the correct alignment with high precision cannot be determined, in extensively folded structure, yet this procedure improves multiple alignment and hence phylogeny construction. The measurable structural parameters of the molecules are directly used as specific characters to construct a phylogenetic tree. These structures are inferred from the sequence of the nucleotides, often using energy minimization [26]. Molecular morphometrics appears to be complimentary to classical primary sequence analysis in phylogenetic studies as it takes into consideration only the size variations of homologous structural segments and this choice implies that the overall architecture of the molecule remains same among the observed taxa. This method helps in taking into account the regions where multiple alignments are barely reliable because of large number of insertion/deletion operation. Besides, the secondary structures are built on each sequence separately thereby making it unnecessary for a sharp computational sequence analysis. Thus homologous recognizable characters on the secondary structures are easily traced out contrary to finding right counterpart for each nucleotide in every other sequence.

Several patterns of predicted secondary structures of RNA that were constructed from unique ITS sequences from different geographical isolates of *Paragonimus*, provided us with the additional information for correct identification of the species prevalent in the region. The secondary structure analysis of the same data also confirmed the results mentioned for primary sequence analysis. Differences in their topology are only due to the fact that there are variable lengths of the sequences. However, there are difficulties in de fining a distance between two related structures with variable topologies [27]. Following earlier studies *P. skrjabini*, *P. miyazaki*, *P. szechuanensis*, *P. hueitungensis *and *P. veocularis *are considered synonyms; the name of *P. hokuoensis *was proposed for two individual metacercaria of distinctive appearance from southern Yunnan and a number of questions remained unresolved [28]. Nevertheless, because there were inconsistencies in the placement of a few *Paragonimus *species, this study needs to be extended, in order to gain a better understanding of the systematics of this group as well as the evolution of their predicted ITS2 RNA secondary structures.

## Conclusion

The molecular study of the genus *Paragonimus*, which is the most common lung fluke throughout the globe, has gained impetus in the recent times. ITS2 motifs (≤ 50 bp in size) can be considered a promising tool for trematode species identification. RNA secondary structure analysis could be a valuable tool for distinguishing new species and completing *Paragonimus *systematic, more so because ITS2 secondary structure contains more information than the usual primary sequence alignment.

## Competing interests

The authors declare that they have no competing interests.

## Authors' contributions

PKP and VT designed this study, carried out molecular works, phylogenetic analysis and wrote the overall manuscript. DKB designed and performed the computational analysis, interpreted data and assisted in writing the manuscript. LMG carried out molecular work while AC assisted with data interpretation. All authors read and approved the final manuscript.

## Note

Other papers from the meeting have been published as part of *BMC Bioinformatics *Volume 10 Supplement 15, 2009: Eighth International Conference on Bioinformatics (InCoB2009): Bioinformatics, available online at http://www.biomedcentral.com/1471-2105/10?issue=S15.
